# Case report: Asymmetric bilateral deep brain stimulation for the treatment of pantothenate kinase-associated neurodegeneration in a patient: a unique case of atypical PKAN with a novel heterozygous PANK2 mutation

**DOI:** 10.3389/fnhum.2024.1448606

**Published:** 2024-10-16

**Authors:** Guo Hong, Zhongwen Zhang, Peiyi Wang, Guoyang Li, Wenli Zhang, Huahui Zou, Xiaoguang Luo

**Affiliations:** ^1^Department of Neurology, Shenzhen People’s Hospital (The Second Clinical Medical College, Jinan University, The First Affiliated Hospital Southern University of Science and Technology), Shenzhen, China; ^2^Shenzhen Clinical Research Centre for Geriatrics, Shenzhen People’s Hospital, Shenzhen, China; ^3^Department of Neurology, The Affiliated Hospital of Nantong University, Nantong, China; ^4^Department of Anesthesiology, Luohu District People’s Hospital, Shenzhen, China

**Keywords:** pantothenate kinase-associated neurodegeneration (PKAN), PANK2 mutation, tiger’s eye sign, gene mutation, asymmetric bilateral deep brain stimulation treatment

## Abstract

Pantothenate kinase-associated neurodegeneration (PKAN) is a rare autosomal recessive hereditary neurodegenerative disorder, usually caused by mutations in the pantothenate kinase 2 (PANK2) gene. We report a young female patient with atypical PKAN, harboring a novel heterozygous PANK2 mutation, diagnosed through clinical imaging and genetic analysis. The patient presented with dystonia and motor dysfunction after onset, but early brain MRI showed normal findings. Due to progressive symptom deterioration, her MRI was reevaluated and the characteristic “eye of the tiger” sign was identified. Further genetic testing revealed that she was a carrier of two heterozygous PANK2 mutations, one being a known pathogenic variant and the other unknown. Given the patient’s clinical presentation, progressive symptoms, and poor response to medication, we boldly attempted asymmetric bilateral deep brain stimulation (abDBS). Postoperative outcomes showed significant symptom improvement. This study suggests that early brain MRI in PKAN patients may not exhibit typical radiological features, leading to potential diagnostic omissions. Furthermore, it highlights the potential therapeutic effect of abDBS in atypical PKAN, particularly in patients with novel heterozygous PANK2 mutations. Asymmetric bilateral deep brain stimulation may represent a promising treatment approach.

## Introduction

Pantothenate kinase-associated neurodegeneration (PKAN) is a rare autosomal recessive inherited neurodegenerative disease characterized by dystonia, extrapyramidal symptoms, and cognitive impairment (Hayflick et al., 2003). This disease was first described in 1922 by two German physicians, Hallervorden and Spatz, hence its alternative name, Hallervorden–Spatz disease (HSD) (Hogarth et al., 2017). PKAN is the most common form of neurodegeneration with brain iron accumulation (NBIA), with an estimated incidence of 1–3 per million, accounting for approximately half of NBIA cases (Hogarth et al., 2017). The disease results from mutations in the pantothenate kinase 2 (PANK2) gene. Based on age of onset and disease progression, PKAN can be classified into two subtypes: classic and atypical (Hayflick et al., 2003). Classic PKAN typically begins at an early age, progresses rapidly, has a poor prognosis, and often leads to early death. In contrast, atypical PKAN presents later in life, progresses more slowly, and has a relatively better prognosis. The diagnosis of PKAN is typically based on clinical symptoms, imaging, and genetics. This report presents a rare case of atypical PKAN with a novel heterozygous PANK2 mutation, demonstrating significant improvement in severe dystonia and motor dysfunction following asymmetric bilateral deep brain stimulation therapy.

## Case presentation

A 26-year-old young Asian woman presented to our institution in late November 2023 with a history of walking difficulties for over 5 years. She had undergone an artificial abortion in 2015 with no other underlying conditions but had a history of vaping for over 6 years. Since the onset of her illness, the patient experienced a weight loss of approximately 20 kg, chronic psychological depression, frequent crying, and mental lethargy. She had been on a long-term liquid diet, with minimal food intake in the week before admission. Additionally, she suffered from poor sleep quality daily, although her bowel movements remained normal.

**Figure 1:**
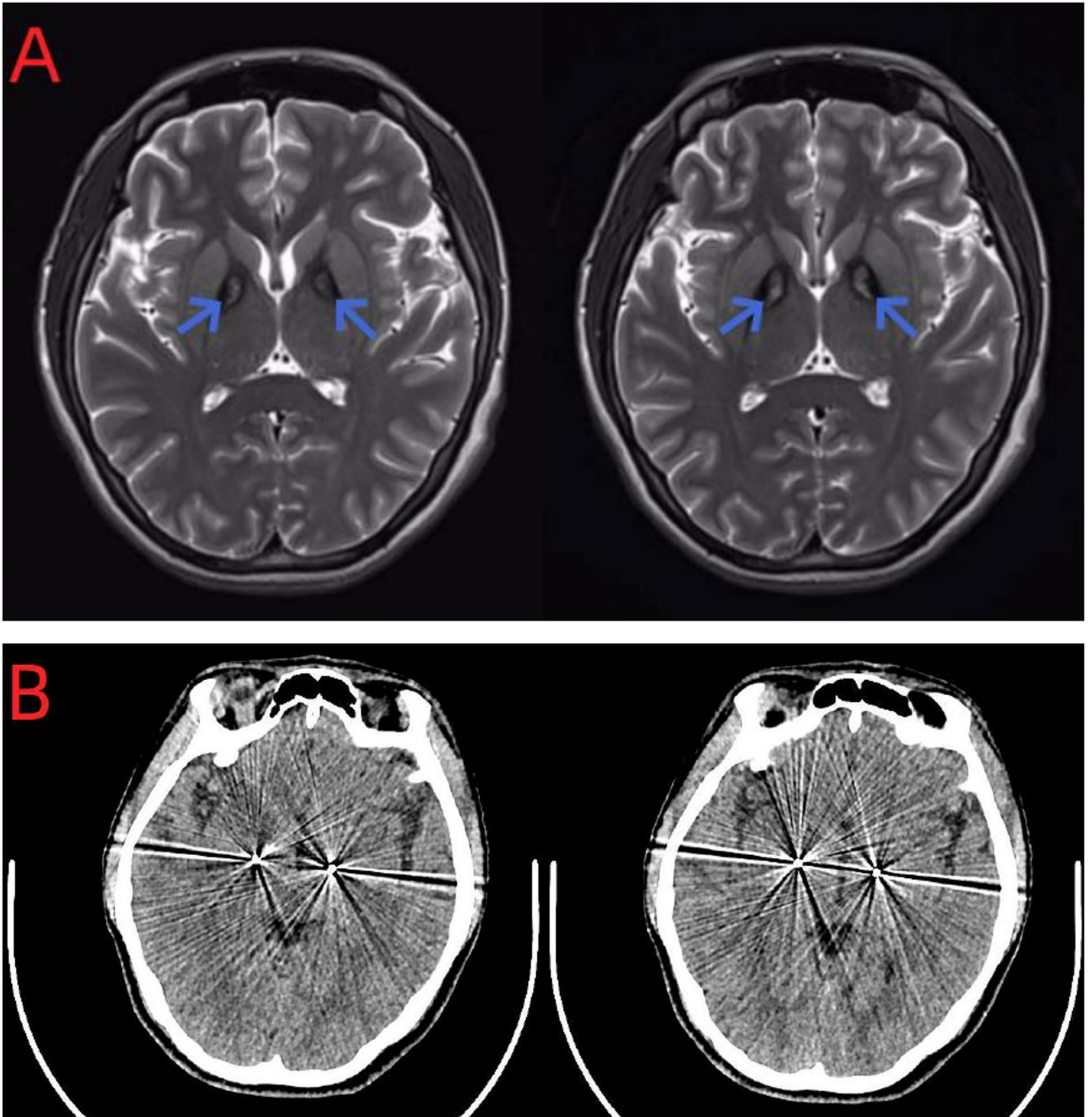
**(A)** Axial brain MRI of the patient. MRI T2WI showed the “tiger eye” sign. **(B)** CT showed the implantation site of the permanent DBS electrode (Medtronic, United States).

**Figure 2:**
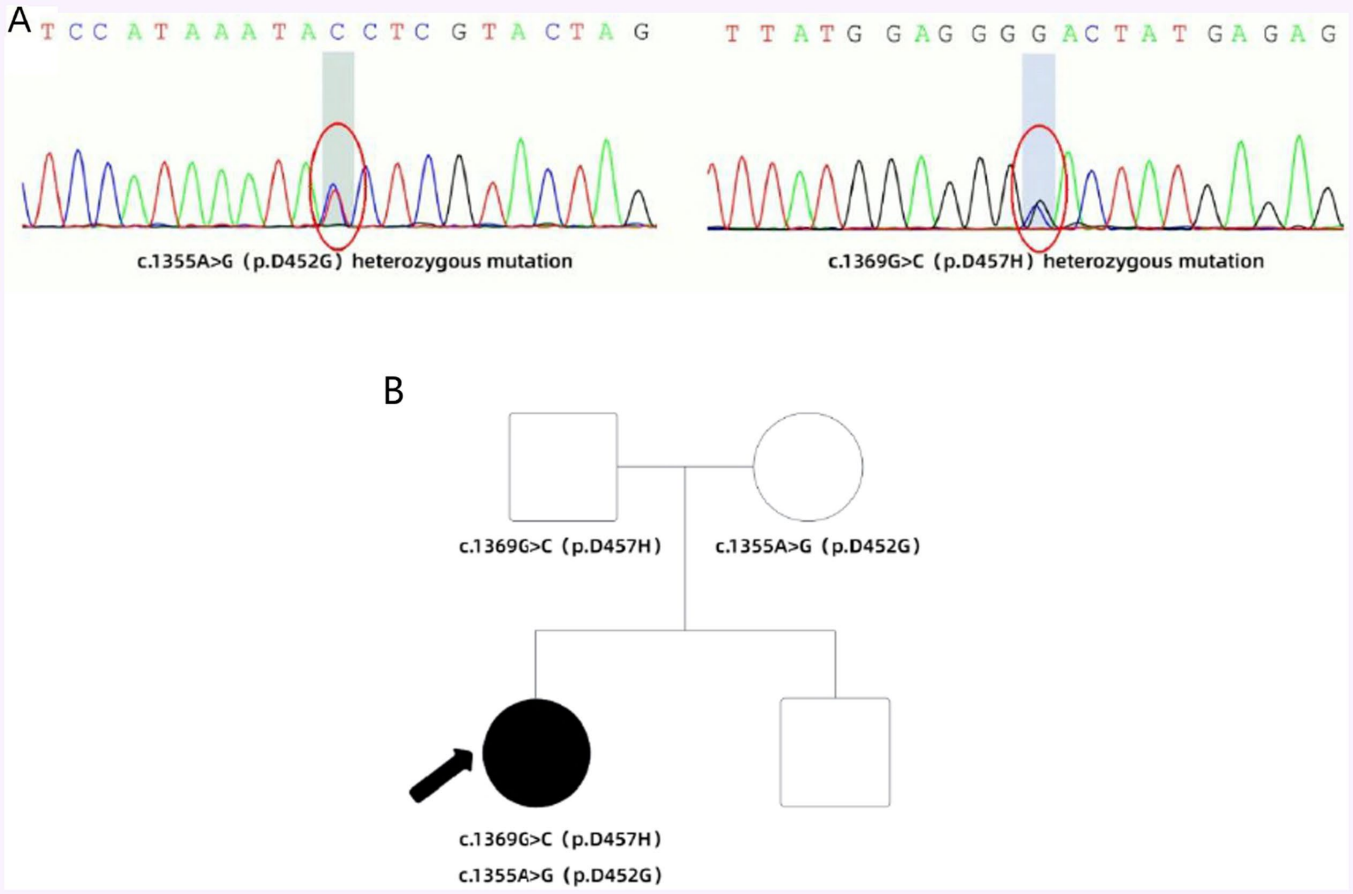
**(A)** According to the guidelines of the American College of Medical Genetics and Genomics (ACMG), genetic testing revealed two heterozygous mutations in the PANK2 gene: a pathogenic mutation c.1355A>G (p.D452G) and a novel mutation c.1369G>C (p.D457H). **(B)** Family with compound heterozygous PANK2 gene mutations. Proband PKAN is indicated with an arrow. Square symbol = male; circle = female; filled symbol = affected.

In early July 2018, the patient experienced a sudden backward tilt of the lower back while bathing. By early September, a stroke-like gait was observed, with the patient’s entire body leaning rightward and backward. The patient could maintain balance while walking and did not experience symptoms such as falling. Later that month, involuntary tremors developed in the right upper limb during both movement and rest. The patient sought medical attention at a local healthcare institution and underwent a cranial MRI scan, which showed no significant abnormalities. Subsequently, symptomatic treatment with medications such as flupentixol, benztropine, and levodopa was initiated, but the symptoms did not significantly improve. By mid-April 2021, the patient’s physical symptoms had progressively worsened, with frequent episodes of tilting the head back while sitting by the bed, occasionally accompanied by convulsions. During episodes of increased convulsions, EEG recordings did not reveal any abnormal, excessive, or synchronized neuronal activity, and the patient remained conscious, excluding typical epileptic seizures. Conversely, the patient’s serum CK levels were elevated, and electromyographic recordings showed repeated involuntary muscle contractions, further suggesting that muscle spasms might be the primary cause of the convulsions. Due to the outbreak of the COVID-19 pandemic, the patient has been resting at home ever since. In early October 2022, the patient experienced muscle stiffness in the lower back and upper body, with certain muscles appearing tense. Lying flat caused extreme tension in the lower back muscles, resulting in an arched posture. Additionally, there was mild difficulty in chewing. By early June 2023, the patient began experiencing difficulty opening the mouth and tight closure of the jaw, without drooling. Swallowing difficulties, accompanied by coughing, necessitated a liquid diet. Moreover, when tilting the neck to the right, the frequency and severity of convulsions increased, accompanied by transient chest tightness and shortness of breath. The patient subsequently sought treatment again at a local medical institution, and his condition did not improve significantly. In late November, for further diagnosis and treatment, the patient came to our hospital. The patient presented with facial muscle tension and inability to eat due to tight closure of the jaw. On the day of admission, the patient received botulinum toxin injections into the facial muscles. The patient’s symptoms were continuously evaluated within 3 weeks after the injections, but unfortunately, no significant improvement was observed. Physical examination upon admission revealed difficulty eating, slow movements, clear consciousness, and dysarthria. The pupils were equal in size and round, the corneal K-F rings were negative, eye movements were normal, and light reflexes were sensitive. There was no nystagmus in both eyes. The nasolabial folds were symmetrical, and tongue protrusion was centered. Muscle tone in the neck and all limbs was elevated, with higher muscle tone on the left side compared to the right side. Muscle strength in all limbs was normal. Deep tendon reflexes were hyperactive (++), and muscle tone was elevated in the lower back, causing an arched posture. The left finger-to-nose test was unsteady with intention tremor. Pathological reflexes were not elicited, and there were no signs of meningeal irritation. Sensory examination was normal. The Unified Dystonia Rating Scale (UDRS) score was 38. Laboratory examination revealed that all blood biochemical indicators were within the normal range. Follow-up MRI of the head revealed symmetric short T2 signals in the bilateral globus pallidus, with long T2 signals within, presenting the typical “eye of the tiger” sign (Figure 1A), suggestive of pantothenate kinase-associated neurodegeneration (PKAN). According to the guidelines of the American College of Medical Genetics and Genomics (ACMG), genetic testing revealed two heterozygous mutations in the PANK2 gene: a pathogenic mutation c.1355A>G (p.D452G) and a novel mutation c.1369G>C (p.D457H). The Sanger sequencing image of the tested loci is shown in Figure 2A. Family verification results indicated that the patient’s father carried the same heterozygous mutation at the c.1369G>C locus, while the patient’s mother carried the same heterozygous mutation at the c.1355A>G locus. However, the patient’s brother did not carry these mutations (Figure 2B). Based on the patient’s medical history, imaging findings, and genetic testing results, the final diagnosis was pantothenate kinase-associated neurodegeneration (PKAN), classified as non-classical type. Considering the patient’s severe dystonia, motor dysfunction, and lack of response to medication, asymmetric bilateral deep brain stimulation surgery was performed under general anesthesia in early December 2023, with the patient’s and family’s consent (Figure 1B). At the same time, the patient and his family were clearly informed before the patient was treated with abDBS that the use of the treatment in this case was off-label, and the possible risks and expected benefits were explained in detail.

**Figure 3:**
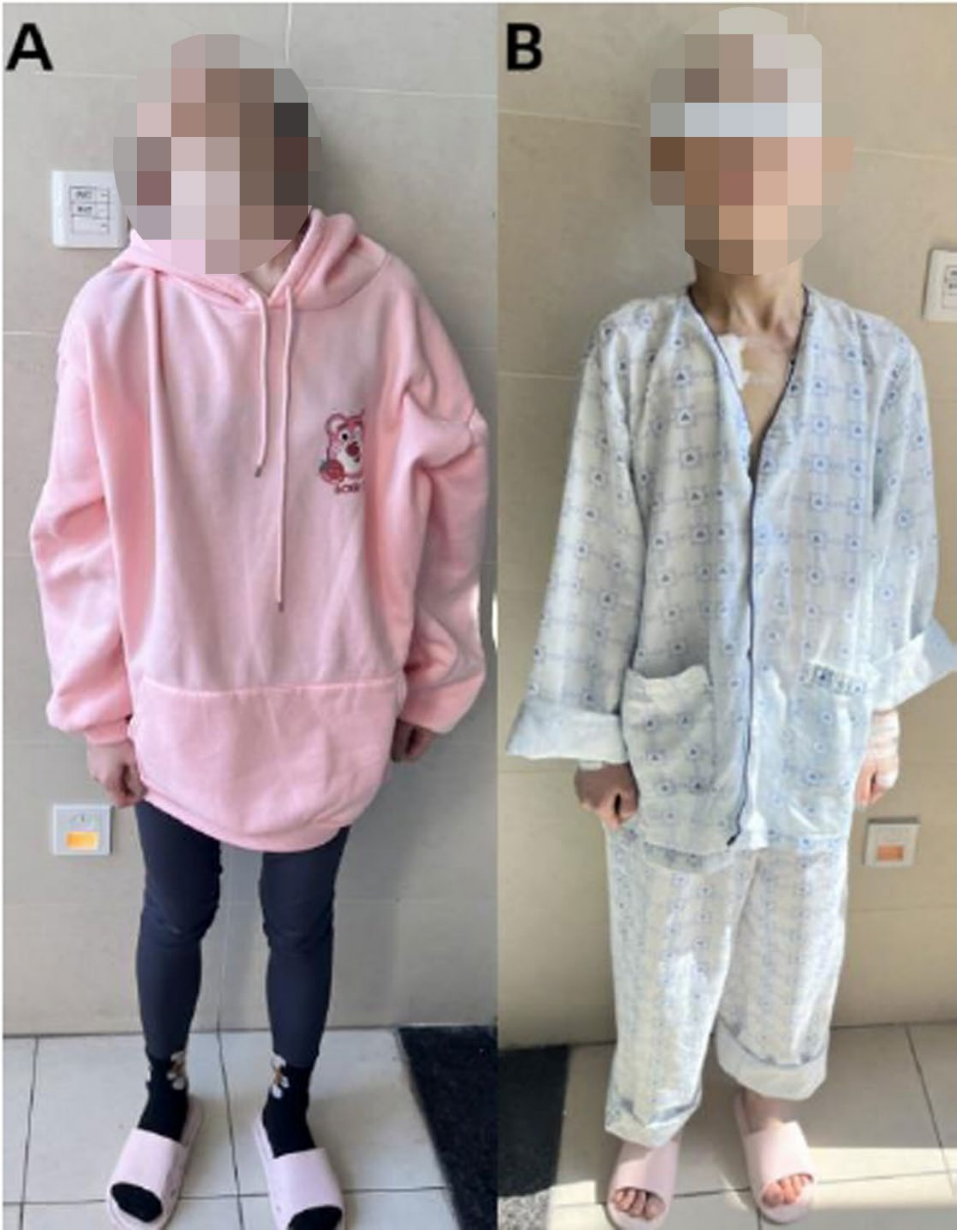
**(A)** Preoperatively, the patient exhibited high muscle tone in the neck and limbs, an abnormal standing posture, and the left sternocleidomastoid muscle was highly developed due to prolonged abnormal contraction. **(B)** Postoperatively, the patient showed a significant reduction in muscle tone in the neck and limbs, allowing for a normal upright standing posture.

The neurostimulator was activated on the day of surgery. Parameters were set to optimize control of dystonia and motor dysfunction without side effects. Based on the patient’s feedback, deep brain stimulation (DBS) was activated using standard settings: right globus pallidus internus (R-GPi)—frequency 130 Hz, pulse width 90 ms, voltage 2.5 V; left subthalamic nucleus (L-STN)—frequency 130 Hz, pulse width 60 ms, voltage 2.5 V. After 1 week of DBS treatment, the patient was able to open their mouth to eat, and the muscle tone in the neck and limbs significantly decreased, allowing them to stand upright (Figure 3). Additionally, there was a noticeable improvement in mental symptoms and sleep quality. The UDRS score at discharge was 17, indicating a 55.3% improvement. The patient is currently under continuous follow-up.

## Discussion

Pantothenate kinase-associated neurodegeneration (PKAN) is a neurodegenerative disease characterized by iron deposition in the basal ganglia and is a subtype of neurodegeneration with brain iron accumulation (NBIA). This autosomal recessive genetic disorder is caused by mutations in the PANK2 gene located on chromosome 20p12.3–13 (Hayflick et al., 2003). Mutations in the PANK2 gene can lead to functional defects or loss of pantothenate kinase 2, thereby affecting coenzyme A synthesis. Coenzyme A deficiency leads to metabolic disturbances within the mitochondria, including impaired fatty acid oxidation and iron deposition. These metabolic disturbances can impair neurons, especially those in the basal ganglia, leading to the clinical symptoms of PKAN (Yang et al., 2022). The clinical manifestations of this disease are primarily characterized by extrapyramidal symptoms, exhibiting significant clinical and genetic heterogeneity (Razmeh et al., 2018). Early-onset classic PKAN typically begins in childhood or adolescence, with symptoms worsening over time. This type typically occurs before the age of 10, with approximately 90% of cases occurring between the ages of 3 and 6 (Hogarth et al., 2017). The initial symptoms primarily include gait disorders and postural abnormalities, characterized by progressive dystonia and dysarthria. Patients may also experience cholecystitis, epilepsy, psychiatric symptoms, retinitis pigmentosa (RP) and optic atrophy (Hogarth et al., 2017). Late-onset non-classic PKAN patients typically develop after age 10, with an average onset around 13 to 14 years, although it can also occur in adulthood (Hayflick, 2006). The main manifestations include dystonia, gait disorders, and unsteady walking. Generally, the later the onset age, the longer the survival period (Hogarth et al., 2017; Hayflick, 2006). Additionally, PKAN exhibits racial variability in clinical manifestations. Lee et al. found that cognitive dysfunction was less common in Asian PKAN patients, who predominantly exhibited dystonia, motor dysfunction, and dysarthria (Lee et al., 2013). Conversely, Caucasian PKAN patients exhibited a more complex clinical presentation, including various psychiatric symptoms, pyramidal tract signs, and parkinsonian features (Lee et al., 2013). The current case involves adult-onset PKAN characterized by dystonia, motor dysfunction, postural abnormalities, and hyperreflexia, aligning with the clinical features of late-onset non-classic Asian PKAN. It also presents clinical characteristics typical of classic PKAN patients, including psychiatric symptoms and sleep disturbances.

Currently, there are no unified diagnostic criteria for PKAN. The diagnosis of this disease generally relies not only on clinical presentation but also on specific auxiliary examinations. Magnetic resonance imaging (MRI) can reveal iron deposition changes in the basal ganglia. In T2-weighted imaging (T2WI), the bilateral globus pallidus exhibits peripheral hypointensity and central punctate hyperintensity, resembling a tiger’s eye, known as the characteristic “eye of the tiger” sign. The presence of this sign is one of the main diagnostic criteria for PKAN. However, not all PKAN patients exhibit this sign, and slightly more than half of the patients can be diagnosed based on the initial MRI findings (Hogarth et al., 2017). Chonillo et al. (2023) observed that the early brain MRI of PKAN patients appears normal, and it is only upon reevaluation of the MRI after symptom deterioration that the subtle “eye of the tiger” sign is identified. This observation is similar to the case of the present case, and it is noteworthy that the patient in this study exhibited a highly typical “eye of the tiger” sign in the reassessed MRI. This highlights the possibility of normal early brain MRI in PKAN, emphasizing the ease of overlooking early-stage PKAN diagnosis. Therefore, repetitive MRI evaluations are crucial for accurate diagnosis. The diagnosis of PKAN is typically suspected based on characteristic symptoms and confirmed through identification of specific mutations in the PANK2 gene. Molecular genetic testing is the most reliable method for confirming the diagnosis of PKAN (Hayflick et al., 2003). Relevant studies have indicated that PANK2 gene mutations are present in both typical PKAN patients and one-third of atypical patients (Hayflick et al., 2003). Currently, over 100 different PANK2 gene mutations have been identified in reported PKAN patients, with missense mutations being the most common. The c.1561G>A mutation is the most frequently observed missense mutation (Kurian and Hayflick, 2013). In this study, the patient was found to have compound heterozygous mutations in the PANK2 gene, both of which are missense mutations. One mutation, c.1355A>G (p.D452G), was inherited from her mother, while the other, c.1369G>C (p.D457H), was inherited from her father. According to the guidelines of the American College of Medical Genetics and Genomics (ACMG) and the gnomAD database, the c.1355A>G mutation is classified as pathogenic with a low population carrier frequency. The c.1369G>C mutation has not been previously reported and is considered a novel mutation, with its pathogenicity yet to be determined. If both mutations are pathogenic, they could theoretically cause the disease, consistent with the autosomal recessive inheritance pattern of PKAN. As for other ancillary tests, such as blood tests and electrophysiological assessments, they lack specificity.

Despite significant advances in the genetic and imaging diagnostics for PKAN, no effective preventive or curative measures exist. The primary focus of treatment is the management of individual symptoms. For focal or segmental dystonia, local botulinum toxin type A injections can be administered (Hefter et al., 2019). For unilateral or generalized dystonia, oral medications such as benzhexol, baclofen, and levodopa/carbidopa combination can be prescribed (Alvarez-Cordoba et al., 2019). These medications can be used individually or in combination. Although these medications offer mild to moderate symptom relief, their therapeutic effects are limited. For drug-resistant PKAN, surgical interventions may need to be considered. Surgical approaches include ablative pallidotomy, thalamotomy, and deep brain stimulation (DBS), with DBS now being the preferred method due to its rapid onset of benefits (Timmermann et al., 2010). Currently, it is widely accepted that the globus pallidus internus (Gpi) target yields better therapeutic outcomes for dystonia, while the subthalamic nucleus (STN) target is more effective for limb tremor and motor dysfunction. Numerous studies have demonstrated the efficacy and safety of Gpi-DBS for PKAN treatment (Hogarth et al., 2017; Svetel et al., 2019; De Vloo et al., 2019; Lim et al., 2012). While most research has focused on pediatric, adolescent patients, and typical cases, some studies have also shown the utility and effectiveness of GPi-DBS in adults and atypical cases. Additionally, STN-DBS has shown improvement in both motor dysfunction and dystonia symptoms among patients (Liu et al., 2017; Ge et al., 2011). A clinical randomized controlled trial from Denmark indicated that after 6 months of bilateral implantation of electrodes in the STN and the GPi in patients with dystonia, the efficacy and safety of STN-DBS were comparable to those of GPi-DBS (Schjerling et al., 2013). Although both STN-DBS and GPi-DBS are viable treatment options for PKAN-related movement disorders, the choice should be made based on the individual patient’s specific symptoms, disease progression, and overall health condition. Considering the unique clinical presentation of the patient in this study, which does not fully meet the typical criteria for PKAN, along with her relatively advanced age and the presence of numerous non-motor symptoms, a tailored approach to selecting DBS targets was necessary. Given the asymmetry of the patient’s symptoms, bilateral GPi or bilateral STN might not provide optimal symptom relief. Clinical evaluation revealed significant limb rigidity, with the left side more affected than the right, and marked hypertrophy of the left sternocleidomastoid muscle due to prolonged dystonia. Clinical evaluation revealed significant limb rigidity, with the left side more affected than the right, and marked hypertrophy of the left sternocleidomastoid muscle due to prolonged dystonia. Therefore, we boldly attempted an asymmetric DBS approach: targeting the GPi on the right to address severe left-sided rigidity and targeting the STN on the left to improve overall motor function and tremor. This strategy aims to provide a more personalized and effective treatment for the patient’s complex and asymmetrical symptoms. Fortunately, following the asymmetric bilateral deep brain stimulation (abDBS) implantation surgery, the patient’s symptoms improved significantly. After 1 week of adjustment therapy, the patient was able to open their mouth to eat, and muscle tone in the neck and limbs was markedly reduced, allowing the patient to stand upright. We also considered the short-term improvement effects of the lesion effect during the early stages of DBS treatment. However, our detailed clinical evaluation revealed that the patient’s symptom improvement was not only significant after 1 week but also persisted during subsequent follow-ups. The sustained symptom improvement following the optimization of DBS parameters further indicates that the improvement was not due to the lesion effect. Moreover, we observed that the brain tissue activity near the DBS electrodes gradually stabilized, further supporting this conclusion. In addition to the improvement in muscle tone, the patient’s psychiatric symptoms and sleep quality also showed significant enhancement. This suggests that abDBS could be a promising treatment option for atypical PKAN patients with novel heterozygous PANK2 mutations. In addition to pharmacological treatment and surgical interventions, lifestyle modifications and dietary adjustments may also aid in managing PKAN. Studies have demonstrated that a ketogenic diet induced disease phenotypes in PKAN mouse models, but these effects could be reversed by pantethine treatment, highlighting the importance of a non-ketogenic diet in PKAN management (Álvarez-Córdoba et al., 2022). Furthermore, pluripotent stem cells derived from PKAN patients have shown significant response to *in vitro* coenzyme A therapy, indicating a potential future treatment approach (Orellana et al., 2016). Overall, due to the complexity and rarity of PKAN, both diagnosis and treatment present significant challenges, necessitating the collaborative efforts of neurologists, geneticists, physical therapists, and other multidisciplinary professionals.

## Conclusion

In conclusion, this study indicates that early brain MRI in PKAN patients may not exhibit typical imaging features, making diagnosis easily overlooked. Additionally, this study demonstrates the potential efficacy of asymmetric bilateral deep brain stimulation (abDBS) in treating atypical PKAN, particularly in patients with novel heterozygous PANK2 mutations. This treatment may represent a promising therapeutic option for these patients.

## Data Availability

The original contributions presented in the study are included in the article/supplementary material, further inquiries can be directed to the corresponding author.

## References

[ref1] Álvarez-Córdoba M. Reche-López D. Cilleros-Holgado P. Talaverón-Rey M. Villalón-García I. Povea-Cabello S.. (2022). Therapeutic approach with commercial supplements for pantothenate kinase-associated neurodegeneration with residual PANK2 expression levels. Orphanet J. Rare Dis. 17:311. doi: 10.1186/s13023-022-02465-9, PMID: 35945593 PMC9364590

[ref2] Alvarez-Cordoba M. Villanueva-Paz M. Villalón-García I. Povea-Cabello S. Suárez-Rivero J. M. Talaverón-Rey M.. (2019). Precision medicine in pantothenate kinase-associated neurodegeneration. Neural Regen. Res. 14, 1177–1185. doi: 10.4103/1673-5374.251203, PMID: 30804242 PMC6425824

[ref3] Chonillo C. C. Rubin B. Rhinehart R. Houghton D. (2023). A case of pantothenate kinase-associated neurodegeneration (PKAN) in a patient with one known pathologic variant and one variant of unknown significance (P11-4.006). Neurology 100:2619. doi: 10.1212/WNL.0000000000202676

[ref4] De Vloo P. Lee D. J. Dallapiazza R. F. Rohani M. Fasano A. Munhoz R. P.. (2019). Deep brain stimulation for pantothenate kinase‐associated neurodegeneration: a meta‐analysis. Mov. Disord. 34, 264–273. doi: 10.1002/mds.27563, PMID: 30633810

[ref5] Ge M. Zhang K. Ma Y. Meng F. G. Hu W. H. Yang A. C.. (2011). Bilateral subthalamic nucleus stimulation in the treatment of neurodegeneration with brain iron accumulation type 1. Stereotact. Funct. Neurosurg. 89, 162–166. doi: 10.1159/000323374, PMID: 21494068

[ref6] Hayflick S. J. (2006). Neurodegeneration with brain iron accumulation: from genes to pathogenesis. Semin. Pediatr. Neurol. 13, 182–185. doi: 10.1016/j.spen.2006.08.007 17101457

[ref7] Hayflick S. J. Westaway S. K. Levinson B. Zhou B. Johnson M. A. Ching K. H. L.. (2003). Genetic, clinical, and radiographic delineation of Hallervorden–Spatz syndrome. N. Engl. J. Med. 348, 33–40. doi: 10.1056/NEJMoa020817, PMID: 12510040

[ref8] Hefter H. Rosenthal D. Bigalke H. Moll M. (2019). Clinical relevance of neutralizing antibodies in botulinum toxin long-term treated still-responding patients with cervical dystonia. Ther. Adv. Neurol. Disord. 12:1278113646. doi: 10.1177/1756286419892078, PMID: 31897089 PMC6918489

[ref9] Hogarth P. Kurian M. A. Gregory A. Csányi B. Zagustin T. Kmiec T.. (2017). Consensus clinical management guideline for pantothenate kinase-associated neurodegeneration (PKAN). Mol. Genet. Metab. 120, 278–287. doi: 10.1016/j.ymgme.2016.11.004, PMID: 28034613

[ref10] Kurian M. A. Hayflick S. J. (2013). Pantothenate kinase-associated neurodegeneration (PKAN) and PLA2G6-associated neurodegeneration (PLAN): review of two major neurodegeneration with brain iron accumulation (NBIA) phenotypes. Int. Rev. Neurobiol. 110, 49–71. doi: 10.1016/B978-0-12-410502-7.00003-X, PMID: 24209433 PMC6059649

[ref11] Lee C. H. Lu C. S. Chuang W. L. Yeh T. H. Jung S. M. Huang C. L.. (2013). Phenotypes and genotypes of patients with pantothenate kinase-associated neurodegeneration in Asian and Caucasian populations: 2 cases and literature review. Sci. World J. 2013:860539. doi: 10.1155/2013/860539, PMID: 24348190 PMC3854131

[ref12] Lim B. C. Ki C. S. Cho A. Hwang H. Kim K. J. Hwang Y. S.. (2012). Pantothenate kinase-associated neurodegeneration in Korea: recurrent R440P mutation in PANK2 and outcome of deep brain stimulation. Eur. J. Neurol. 19, 556–561. doi: 10.1111/j.1468-1331.2011.03589.x, PMID: 22103354

[ref13] Liu Z. Liu Y. Yang Y. Wang L. Dou W. Guo J.. (2017). Subthalamic nuclei stimulation in patients with pantothenate kinase-associated neurodegeneration (PKAN). Neuromodulation 20, 484–491. doi: 10.1111/ner.12549 28055131

[ref14] Orellana D. I. Santambrogio P. Rubio A. Yekhlef L. Cancellieri C. Dusi S.. (2016). Coenzyme a corrects pathological defects in human neurons of PANK2-associated neurodegeneration. EMBO Mol. Med. 8, 1197–1211. doi: 10.15252/emmm.201606391, PMID: 27516453 PMC5048368

[ref15] Razmeh S. Habibi A. H. Orooji M. Alizadeh E. Moradiankokhdan K. Razmeh B. (2018). Pantothenate kinase-associated neurodegeneration: clinical aspects, diagnosis and treatments. Neurol. Int. 10:7516. doi: 10.4081/ni.2018.7516, PMID: 29844889 PMC5937219

[ref16] Schjerling L. Hjermind L. E. Jespersen B. Madsen F. F. Brennum J. Jensen S. R.. (2013). A randomized double-blind crossover trial comparing subthalamic and pallidal deep brain stimulation for dystonia. J. Neurosurg. 119, 1537–1545. doi: 10.3171/2013.8.JNS13844, PMID: 24116723

[ref17] Svetel M. Tomić A. Dragašević N. Petrović I. Kresojević N. Jech R.. (2019). Clinical course of patients with pantothenate kinase-associated neurodegeneration (PKAN) before and after DBS surgery. J. Neurol. 266, 2962–2969. doi: 10.1007/s00415-019-09499-3 31463603

[ref18] Timmermann L. Pauls K. A. Wieland K. Jech R. Kurlemann G. Sharma N.. (2010). Dystonia in neurodegeneration with brain iron accumulation: outcome of bilateral pallidal stimulation. Brain 133, 701–712. doi: 10.1093/brain/awq022, PMID: 20207700 PMC2842517

[ref19] Yang D. Cho S. Cho S. I. Kim M. Seong M. W. Park S. S. (2022). Genetic mutation spectrum of pantothenate kinase-associated neurodegeneration expanded by breakpoint sequencing in pantothenate kinase 2 gene. Orphanet J. Rare Dis. 17:111. doi: 10.1186/s13023-022-02251-7, PMID: 35246191 PMC8896100

